# Employment of Self-Adaptive Bayesian Neural Network for Systematic Antenna Design: Improving Wireless Networks Functionalities

**DOI:** 10.3390/mi14030594

**Published:** 2023-03-02

**Authors:** Khaled Aliqab, Muhammad Ammar Sohaib, Farman Ali, Ammar Armghan, Meshari Alsharari

**Affiliations:** 1 Department of Electrical Engineering, College of Engineering, Jouf University, Sakaka 72388, Saudi Arabia; 2 Department of Electrical Engineering, Qurtuba University of Science and IT, Dera Ismail Khan 29050, KP, Pakistan

**Keywords:** antenna computation time, optimized antenna pattern, self-adaptive Bayesian neural network, wireless network

## Abstract

The performance of wireless networks is related to the optimized structure of the antenna. Therefore, in this paper, a Machine Learning (ML)-assisted new methodology named Self-Adaptive Bayesian Neural Network (SABNN) is proposed, aiming to optimize the antenna pattern for next-generation wireless networks. In addition, the statistical analysis for the presented SABNN is evaluated in this paper and compared with the current Gaussian Process (GP). The training cost and convergence speed are also discussed in this paper. In the final stage, the proposed model’s measured results are demonstrated, showing that the system has optimized outcomes with less calculation time.

## 1. Introduction

In antenna design, evolutionary algorithms (EAs) are frequently employed. They demonstrate advantages for various design instances due to their capacity to break out of local optima without the need for an initial design and generality. EA-driven antenna design is arguably dominated by particle swarm optimization (PSO) and differential evolution (DE) algorithms. The optimization time, however, can be prohibitive given that full-wave electromagnetic (EM) simulations are frequently required to acquire correct performance of a candidate design and that EAs frequently need tens of thousands of such EM simulations to obtain the best design [1,2,3]. Printed antennas are usually utilized in numerous radar systems and communications applications because of effortless and straightforward integration with transceivers, low cost, and low profile. While in the frequency range associated with 13.5 GHz intended for the particular Broadcast Satellite Services (BSS), there is always the massive requirement of microstrip arrays [4,5,6,7]. The 77 GHz frequency band is inclining for automobile radar purposes. Inspired by this tendency, the on-chip antennas that happen to be appropriate for CMOS radios in gigahertzes have also turned out to be feasible, since the millimeter wavelength enables the implementation of numerous antennas on a single chip [8]. The advantages associated with additive manufacturing (AM) techniques, including 3D printing, cause it to become achievable for every design having complicated interior and exterior geometry being manufactured instantly by a computer-aided design (CAD). The significance associated with 3D-printed heterogeneous substrates are analyzed in [9], and a significant improvement in bandwidth is observed. In addition, the air-cored substrates are reviewed, as well as examined.

The MillimeterWave (mmW) has been previously utilized in RADAR systems and satellite communication. These waves can undoubtedly travel through longer ranges in cases where Line of Sight (LoS) is made available; however, without having LoS, they cannot travel several hundreds of meters. Excessive propagation impairment is mainly because the atmospheric absorption of waves should be considered while designing an antenna for mmW [10]. The assimilation by oxygen molecules may cause incredibly huge attenuation [6]. This particular design will be suitable for the frequency spectrum of V-band ranges from 50 GHz to 75 GHz communication, which is certainly intended for applications associated with short-range communication. The need regarding high gain for 5th Generation communication leads this work to design an optimum antenna, which can be widely recognized because of its highly uncomplicated composition and excessive growth. The previously designed Yagi antenna with high yield and high directive beams was complex in structure, complex in fabrication, bulky in body weight, and significant in dimensions. The several microstrip Yagi antennas having particular radiation mechanisms are usually recommended to acquire a highly directive beam [11]. Yagi antennas hold various positive aspects for mobile communication systems over other commonly utilized antennas [12]. The initial imprinted Yagi antenna is constructed from four patches: the driven element and the reflector elements, along with two director elements that have been electromagnetically connected to generate a high directive beam [9]. As mentioned prior, the antenna’s compact size is a terrific consideration because the antenna is intended for being implemented inside portable devices.

The outcomes related to a modification in the substrate materials designed for 60 GHz V-band highly directive Yagi antenna have been introduced in [10]. Intended for Local Positioning Systems (LPS) applications, one particular multilayered stacked quasi-Yagi antenna with high gain had been designed as well as examined at the frequency of 5.8 GHz [13]. However, the models ended up excessively bulky and oversized in dimensions. In [14], another highly directional beam antenna design is recommended. On the other hand, it appeared heavy in bodyweight due to its different parasitic substrate on top of the innovation. However, an additional technique is exercised in [15], which still utilized a slotted ground of rectangular shape because its complication in production is not a suitable fit. In [16], a multiband high-gain antenna is suggested; however, having minimal bandwidth makes it inappropriate. In [17], to achieve a high gain, a microstrip Yagi antenna is offered, still with Periodic Bandgap (PBG), because its complication in structure design makes it unsuitable.

Similarly, a Resonant Cavity Antenna (RCA) intended for the high-gain dual band is proposed in [18], in which stepped rings are added to improve gain and bandwidth. The additional enhancement in progress is certainly accomplished by using a Partially Reflective Surface (PRS), owning to the high permittivity substrate and circular patch etched on it. An innovative approach regarding feeding the dielectric resonator working with a metallic round patch antenna intended for mmW frequency band is suggested in [19].

Porcelain material based upon a rectangle-shaped Dielectric Resonator Antenna (DRA) is placed over the substrate with relatively low permittivity and fed by a metallic circular patch. The development investigation and analysis are carried out using a cross-slot aperture and rectangular slot. By implementing a cross-slot gap on the ground plane, a substantial improvement in gain along with bandwidth has been accomplished. The singly fed dielectric DRA recommended in this article offers high growth, efficiency, and wide bandwidth.

A planar antenna with a high-gain-having conical beam intended for drone applications of mmW is radiated by utilizing some simple monopole [20] exclusively. Moreover, the operation and overall performance of the suggested design have been explored on top of a metallic, electrically significant plate. Among the particular side reflections and source radiation having the in-phase state, the suggested structure presented decent Conical Beam Radiation (CBR) efficiency. However, additionally attained increased peak gain and high radiation efficiency are in contrast with various other described designs on Conical Beam (CB) planar antennas having uncomplicated structure. The suggested planar antenna with high-gain-having CB is investigated along with various other described designs in expressing the beam-pointing angles, impedance BWs, radiation efficiencies, peak gains, dimensions, and planar/nonplanar geometries.

In contrast with various other designs on CB planar antennas, the suggested model accomplishes the maximum gain and maximum radiation efficiency, having a relatively decent 10 dB impedance bandwidth. The proposed model in [21] comprises about three distinct models of array antennas primarily based on microstrip. To obtain resonance frequencies associated with the THz band, we applied corporate-series-fed approaches completely modeled using a CST MWS solver. The Substrate-Integrated Waveguide (SIW) is used for two proposed models. In contrast, Frequency-Selective Surfaces (FSSs) are utilized by the third proposed model that is undoubtedly intended to increase the actual antenna gain even more.

R. Rehman et al. in [22] proposed a planar antenna with high gain having two ports intended for satellite band and future 5th Generation mmW. In addition to operating in the mmW spectrum, the suggested model comes with an additional characteristic to operate for that satellite X-band at the same time. The proposed model achieves a considerable and significant gain by taking advantage of an inverse microstrip Yagi director geometry. The maximum gain from the model accomplishes the maximum value to obtain E-plane with much better directivity. The structure possesses a simple planar geometry with an imperfect ground for the satellite X-band, seen giving polarization diversity for being utilized to obtain the WGS satellite system. The Yagi geometry helps make the design attain a significantly higher gain that is demanded, belonging to the mmW band along with considerably better impedance matching and helps it accomplish the additional characteristic to resonate within the satellite X-band at the same time.

In [23], a planar and dual-band Multi-Input Multi-Output (MIMO) mmW structure intended for 5th Generation communication applications was introduced. The dual-band design operating in 27 GHz and 39 GHz consists of monopole elements. The model is undoubtedly fabricated on Rogers 4003C substrate. The design, apart from acquiring a decent radiation efficiency of 98 to 99%, offers a decent mutual coupling among the ports and a maximum gain spread of 5–5.7 dBi. The suggested model has become successful in acquiring a low-profile structure with excellent compactness and a good gain; however, the usage of monopole elements leads to a significantly less directive radiation pattern.

Designing a single wideband antenna with good directivity and gain for the V-Band [24], or 50–75 GHz mmW band, is the primary goal of this research. The proposed design has the benefits of being lightweight, high-gain, somewhat efficient, and broadband. The objective is to significantly reduce the training cost and convergence speed while creating a universal antenna technique with specifications and changeable design characteristics. Therefore, a method for antenna optimization termed Self-Adaptive Bayesian Neural Network surrogate model-assisted differential evolution is proposed. The dimensions of the suggested design are modest.

## 2. Theoretical Investigations for Antenna Design and Proposed Algorithm

The previous section discussed the background and literature of the proposed high-gain antenna design. This section explains the analytical approach for validation of the structure and parameters of the proposed SABNN-based antenna model. At first, the basic principle of currently used techniques such as Gaussian Process (GP) is discussed in this section. Consider the observations m $\left(u=u^{1},\;\ldots u^{n}\right)$ and $\left(v=v^{1},\;\ldots v^{n}\right)$, where the $v\left(u\right)$ is the sample of Gaussian-distributed stochastic process with variance *σ* and mean *µ*. Based on the m observations, the GP predicts the value of $v\left(u\right)$ for next u; this correlation function [25,26,27,28] is written as

(1) $$Corr\left(u^{a}u^{b}\right)=\exp(-\overset{j}{\underset{i=1}{\sum }}\theta_{i}\left|u_{i}^{a}-u_{i}^{b}\right|pi$$

where the dimension of u is represented by j, and pi and $\theta_{i}$ are the hyper parameters. The liklihood function is calculated as

(2) $$\frac{1}{{\left(2\pi \sigma^{2}\right)}^{m/2}\sqrt{\det\left(H\right)}}\exp\left[-\frac{{\left(v-\mu U\right)}^{T}H^{-1}\left(v-\mu U\right)}{2\sigma^{2}}\right]$$

where U is an m×1 vector, and H is m×m covariance matrix. The predicted value and prediction uncertainty $v\left(u^{*}\right)$ and $S\left(u^{*}\right)$ are discussed [29,30] as

(3) $$v\left(u^{*}\right)=\mu +r^{T}H^{-1}\left(v-U\mu \right)$$

The prediction uncertainty plays a key role when judging the potential of user antenna design. For this operation, the prescreening, low confidence bound (LCB), and probability are widely used. The LCB method is introduced in this paper using the objective function $v\left(u\right)$ with $N\left(v\left(u\right),S^{2}\left(u\right)\right)$ predictive distributions. This is written as

(4) $$v_{cd}=y\left(u^{*}\right)-\omega S\left(u\right)$$

where ω is constant; this is set for antenna problems in AI algorithms. The training cost and huge computational time of GP are the main drawbacks. Thus, to handle these constraints, the huge computational time, and training cost, in this paper, the SADE method is proposed for antenna design optimization.

**Figure 1:**
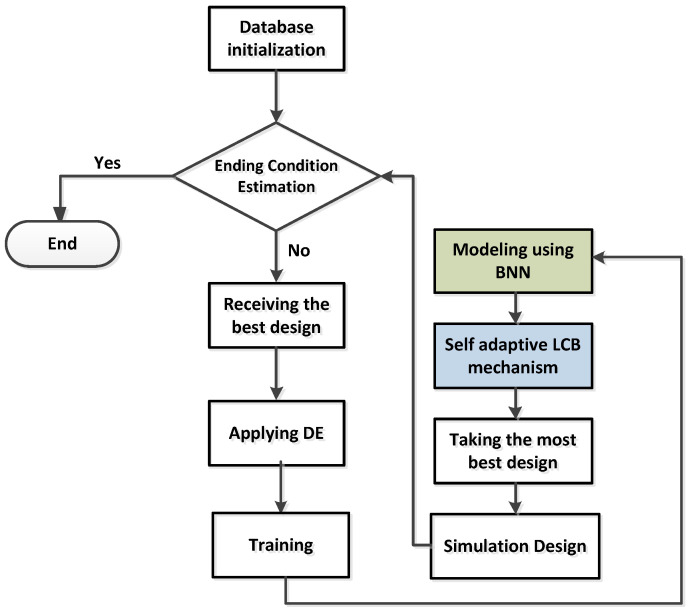
Flow chart description of presented SADE for antenna design optimization.

There are two optimization techniques used for antenna design, named local optimization and global optimization. The global optimization method is applied in this paper for antenna design using an evolutionary algorithm (EA). Taking $\Psi_{pop}$ population, which has $N_{pop}$ solutions, and each solution is denoted by $u=u_{1},\ldots \ldots u_{n}\in H^{*}$, the donor vector for creating a child solution $x=x_{1},\ldots .x_{n}$ is calculated as

(5) $$y^{a}=u^{a}+F.\left(u^{best}-u^{a}\right)+F.\left(u^{r1}-u^{r2}\right)$$

where $u^{a}$ means the $a^{th}$ vector in current $\Psi_{pop},$ $u^{best}$ denotes the best user solution in current $\Psi_{pop},$ $u^{r1}$ and $u^{r2}$ are randomly selected mutual solutions from the population. The parameter $y^{a}$ represents the $a^{th}$ mutual vector and F is used for scaling factor. Figure 1 explores the flow chart of the presented algorithm, which shows the basic role of the BNN and LCB mechanisms.

### Bayesian Neural Network Model for Optimized Antenna Design

**Figure 2:**
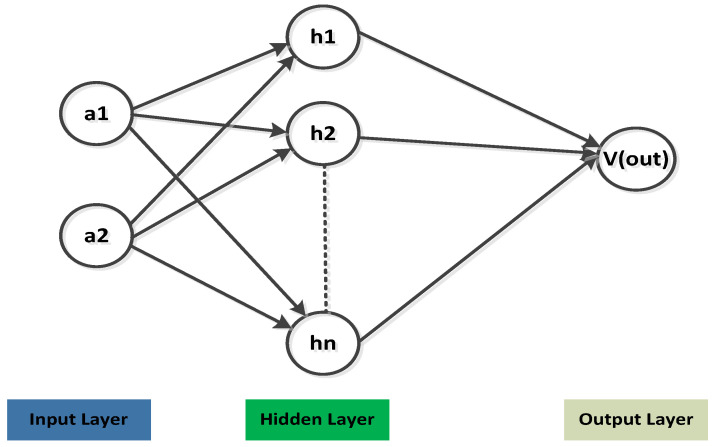
Fundamental structure of the presented BNN model.

To meet the requirements for efficient antenna design, such as minimum training cost and prediction uncertainty for design, as well as provide high-quality predicted value, the Bayesian Neural Network (BNN) is a promising solution. The BNN procedure has valuable contribution towards optimized antenna design. Taking the variable u and v for antenna design while using the ANN approach, the model parameters are written as

(6) $$\theta =w_{1}\ldots \ldots w_{n},\;g_{1}\ldots \ldots .\;g_{m}$$

where g denotes the basis and w is used as the weight. The nonlinear activation function is used in the multilayer ANN model, aiming to transform the linear domain of each layer. The optimized θ is then used for prediction. Comparing with the ANN approach, the BNN-based network structure remains constant, however, the θ becomes a stochastic variable, including $p\left(\theta \right)$ probability distribution. The basic description of the BNN model is declared in Figure 2. Applying the Bayes theorem on input and output training is written as

(7) $$p\left(\frac{\theta }{T_{d}}\right)=\frac{p\left(\frac{T_{dv}}{T_{du}},\theta \right)p\left(\theta \right)}{\int p(\frac{T_{dv}}{T_{du}},\theta )p\left(\theta \right)d\theta }$$

where $p\left(\theta \right)$ and $p\left(\frac{\theta }{T_{d}}\right)$ are the prior and posterior, respectively, and $p\left(\frac{T_{dv}}{T_{du}},\theta \right)$ is the likelihood. The prediction uncertainty and predicted value are achieved by the posterior, which are the objectives of this work.

## 3. Proposed Antenna Framework

**Figure 3:**
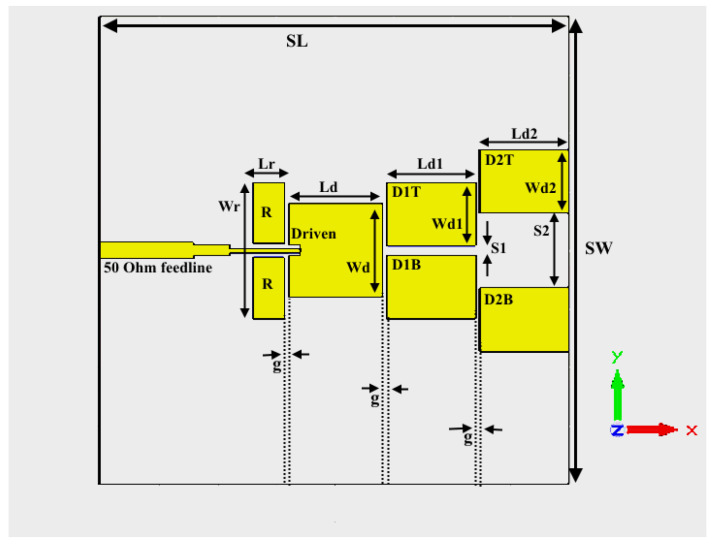
Schematic view of antenna design.

Applying the equations related to the patch antenna presented in the preceding part, the antenna’s rough design is created. A parametric sweep and CST optimizer are used to optimize the parameters and variables. Figure 3 depicts the antenna structure (CST perspective) and the design’s schematic view. A driven element, four directors, two reflector elements, and feeding composition make up the antenna’s seven patch elements. For ease of analysis, we refer to the reflector element “R” as a single element with a 0.24 mm gap through the middle to pass the feedline. A quarter-wave transformer is used to convert the 50 ohm transition feedline utilized in the feeding arrangement into a high-impedance line. The high-impedance line is used to prevent radiation from the feedline near the driven element from interfering with the radiation from the antenna, which would lower gain.

D (Driven element), D1T (Top Director 1), D1B (Bottom Director 1), D2T (Top Director 2), and D2B make up the remaining elements (Bottom Director 2). To simulate the antenna design, a double copper-clad board made of RT/Duroid 5880 material (epsilon = 2.2) was used. The Reflector Element R with Wr and Lr; the Driven Element D with Ld and Wd; the Director 1 element D1T and D1B with Ld1 and Wd1 indicating the value of length as well as width; and the Director 2 element D1T and D2B with Ld2 and Wd2, indicating the length and width, respectively. The length of g separates every element along the x-axis. S1 divides the Top Directors 1 and Bottom Directors 1, while S2 displays the separation between the Top Directors 2 and Bottom Directors 2. SL and SW stand for length and width of the substrate, respectively. The substrate has a thickness of 0.1 mm. The substrate’s overall dimensions are 10.01 × 10.8 mm^2^. The CST MWS design tool simulates the design. The suggested design’s parametric values are shown in mm in Table 1.

## 4. Heterogeneous Substrate and Analysis

Microstrip antennas are often made on a homogeneous substrate and are low-profile, conformal, lightweight, and compact in size. By using a dielectric with a high permittivity value, the size of the antenna can be easily reduced. The energy in the surface wave modes is raised by this rise in dielectric though. Because they are diffracted via the margins of the ground plane of finite size, these surface waves decrease the performance and efficiency of the antenna and cause interference with the radiation pattern. These negative effects might be minimized by the reductions in surface wave modes. The main cause of surface wave radiation for thin-substrate microstrip antennas is unquestionably the TM0 mode, and heterogeneous substrates provide the solution to nearly completely suppress all of the surface waves inspired by this mode.

The substrate surrounding the patch is simply removed, either completely or partially, to create heterogeneous substrates. By suppressing the surface waves, this method has been utilized to boost the gain of a microstrip antenna. The antenna has also undergone substrate removal, which has significantly improved bandwidth and efficiency.

**Figure 4:**
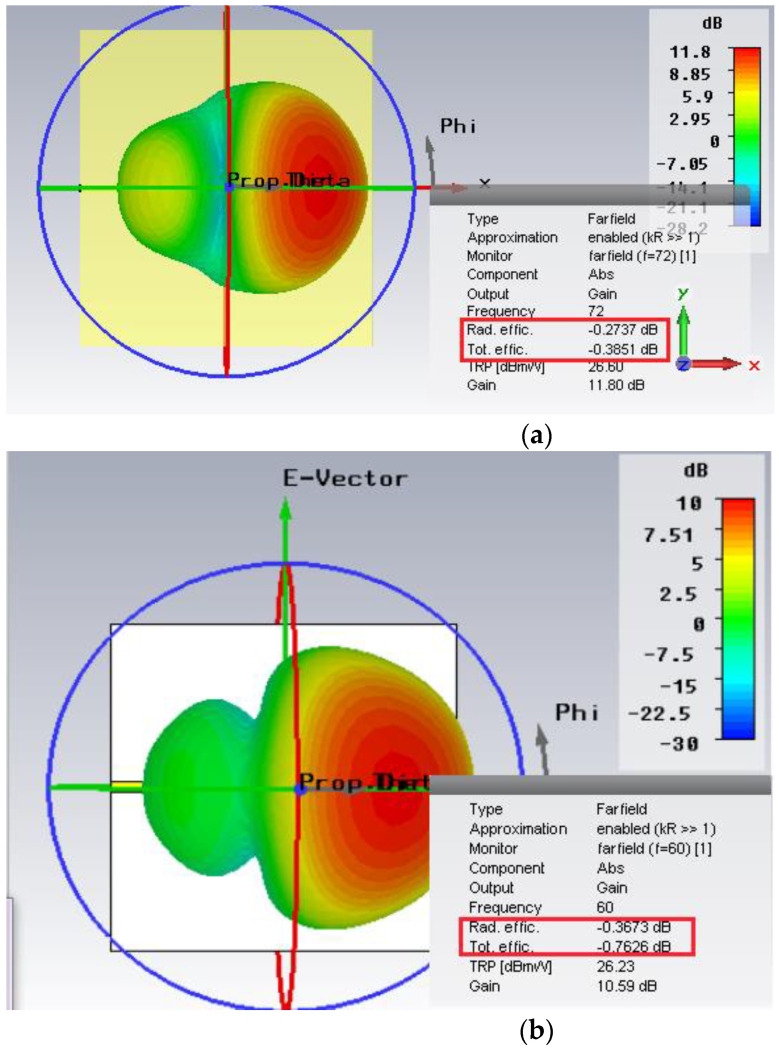
Rad. and tot. efficiency: (**a**) with heterogeneous substrate and (**b**) without heterogeneous substrate.

Therefore, higher gain, bandwidth, and efficiency are due to the heterogeneous substrate, as mentioned in Figure 4. The designed with heterogeneous substrate (Figure 4a) has a radiation efficiency of −0.2737 dB and total efficiency of −0.3851dB. The design without heterogeneous substrate (Figure 4b) has a radiation efficiency of −0.3673 dB and total efficiency of −0.7626 dB. The design with heterogeneous substrate is more efficient as compared with the plain substrate. The patch traces and the piece that was removed from the substrate are an exact match. Prior to the invention of 3D printers, it was impossible to produce this kind of substrate, but now it is possible to precisely construct heterogeneous substrates.

**Figure 5:**
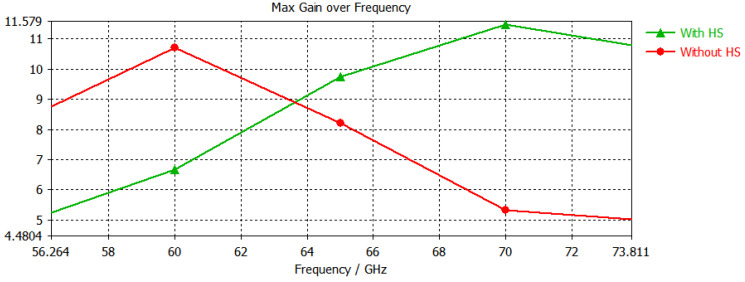
Comparison of with heterogeneous substrate and without heterogenous substrate in terms of frequency and gain.

Figure 5 compares the outcomes of the proposed model based on it with and without heterogeneous substrate. The horizontal axis is associated with the frequency range, while the vertical axis is maximum gain on the specific frequency. The without heterogeneous substrate model has a max. gain of 10.2 and varies sharply with an increase or decrease in frequency, while the design with heterogeneous substrate has a max. gain of 11.8 and is quite stable as compared with the design without heterogeneous substrate.

The gaps in the element which separates every specific component, irrespective of whether it is on the x-axis or even the y-axis, execute a vital function in the layout. The gap ‘g,’ which is undoubtedly on the x-axis, is the gap among all the individual elements along the identical x-axis. This distance is accountable regarding mutual coupling among driven elements and parasitic elements.

**Figure 6:**
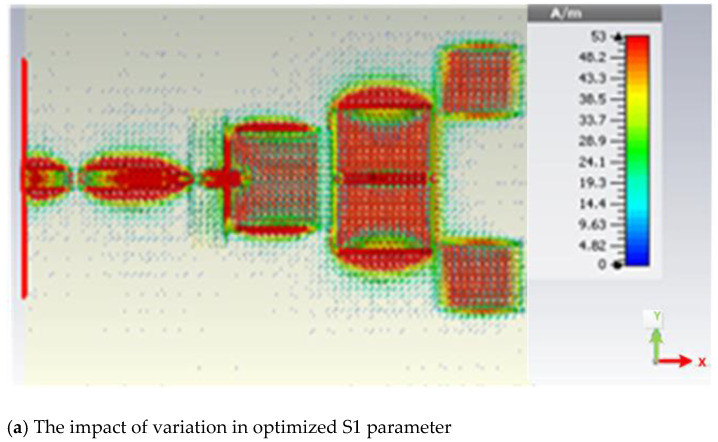

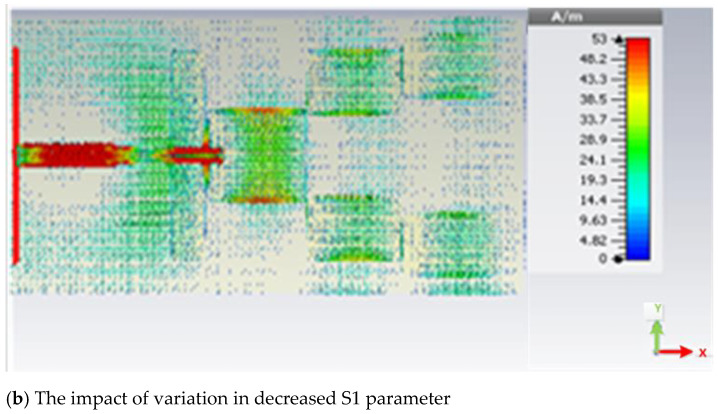
The surface current distribution.

Due to their shorter length than the driven element, the D1 elements (D1T and D1B) are actually in charge of obtaining beam directionality and producing an additional band at a higher frequency. S1 and S2, which represent the gaps between Top Directors and Bottom Directors and are located along the y-axis, are typically in charge of improving. To obtain the strong coupling between the Driven and Director 1 elements, the S1 parameter needs to be small. The distance between the director and driven components must be kept as short as feasible in order to put the parasitic elements next to the driven elements. The gap capacitance will decrease as the electric field coupling increases. The top surface current distribution shown in Figure 4 will make the impact of changing the S1 parameter clearer. The S1 in Figure 6a is in accordance with the plan, showing the excellent surface current distribution caused by good coupling between Director 1 and Driven elements. The S1 parameter is substantially larger than the initial value in Figure 6b, which leads to poor coupling between Driven and Director elements and a reduced surface current distribution.

A single radiating element with a width of ‘2W’ is how the sides of the D1 elements behave. The S2 parameter can be positioned in a variety of ways to produce the largest possible effective aperture. The electrical width of the effective aperture grows as the S2 parameter increases, increasing the gain. The coupling between the D2 and D1 elements decreases when S2 grows too much, which causes gain to drop. The gap capacitance and the fringing capacitance should be adequate for the D2 element to acquire the required radiation if the S2 value is modest enough.

**Figure 7:**
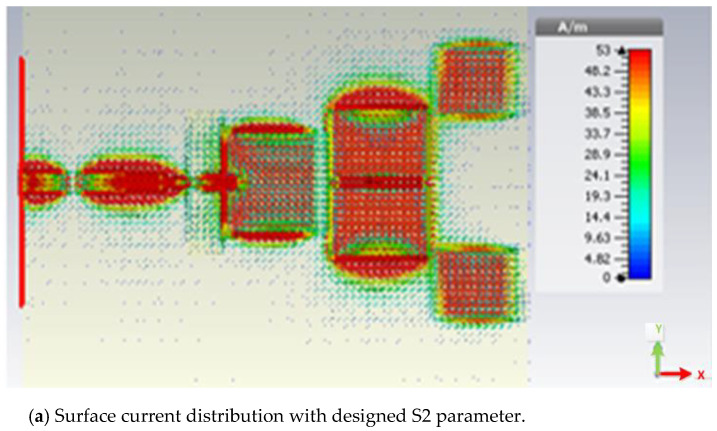

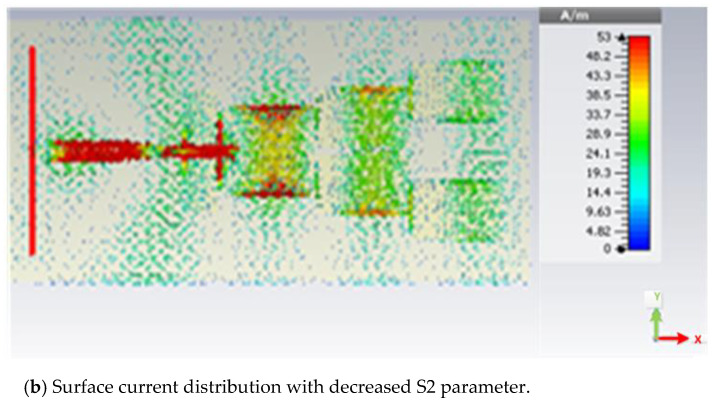
Surface current distribution with S2 parameter.

The effects of changing the S2 parameter up or down are clearly seen in the surface current distribution shown in Figure 7a,b. As previously stated, the gain improvement is caused by the S2 parameter, which widens the antenna’s effective aperture. Director 1 has an effective width that is nearly twice as wide as its physical breadth. To obtain the smallest radiations required for resonance, the center of Director 2 may therefore be placed next to the center of Director 1. Therefore, it was found that raising S2 causes gains to continuously improve, and vice versa. Due to insufficient coupling capacitance, an excessive increase in the S2 parameter will cause yields to drop.

**Figure 8:**
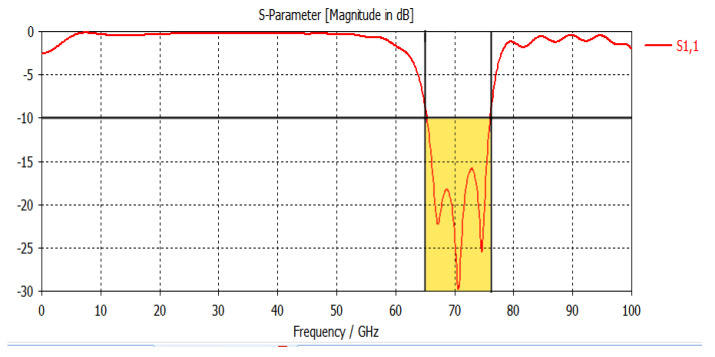
S_11_ parameter of the simulated design.

As shown in Figure 6a, the radiation is at its greatest because D2 elements are connected from the margins of the D1 component. This causes the effective aperture to have a wider electrical field, which increases gain. The antenna’s electrical width is narrower in Figure 6b than in Figure 5a, which causes a weak surface current distribution and lower gain. The design’s simulation results show an impedance bandwidth of 15.17% in a range of 10.72 GHz. In Figure 8, the S11 parameter is displayed.

**Figure 9:**
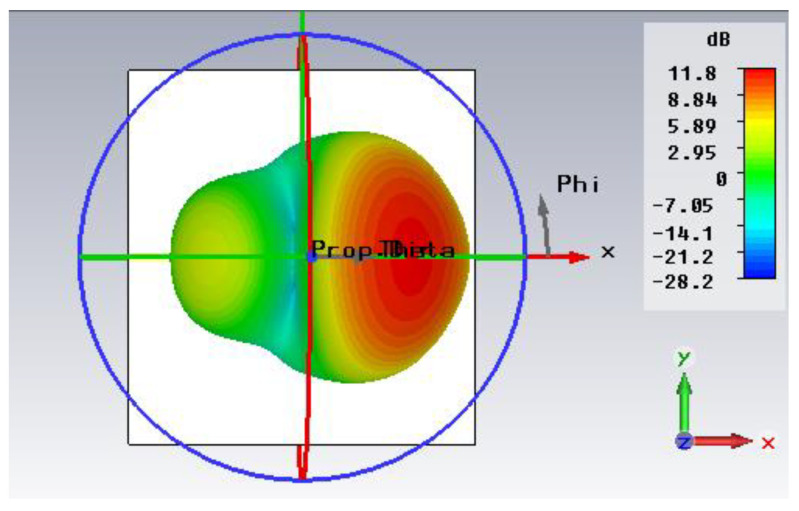
Radiation pattern of far field at 72 GHz.

Figure 9 illustrates the 3D far-field pattern for 72 GHz, which has a high gain of 11.8 dB and a directivity of 12.1 dBi.

**Figure 10:**
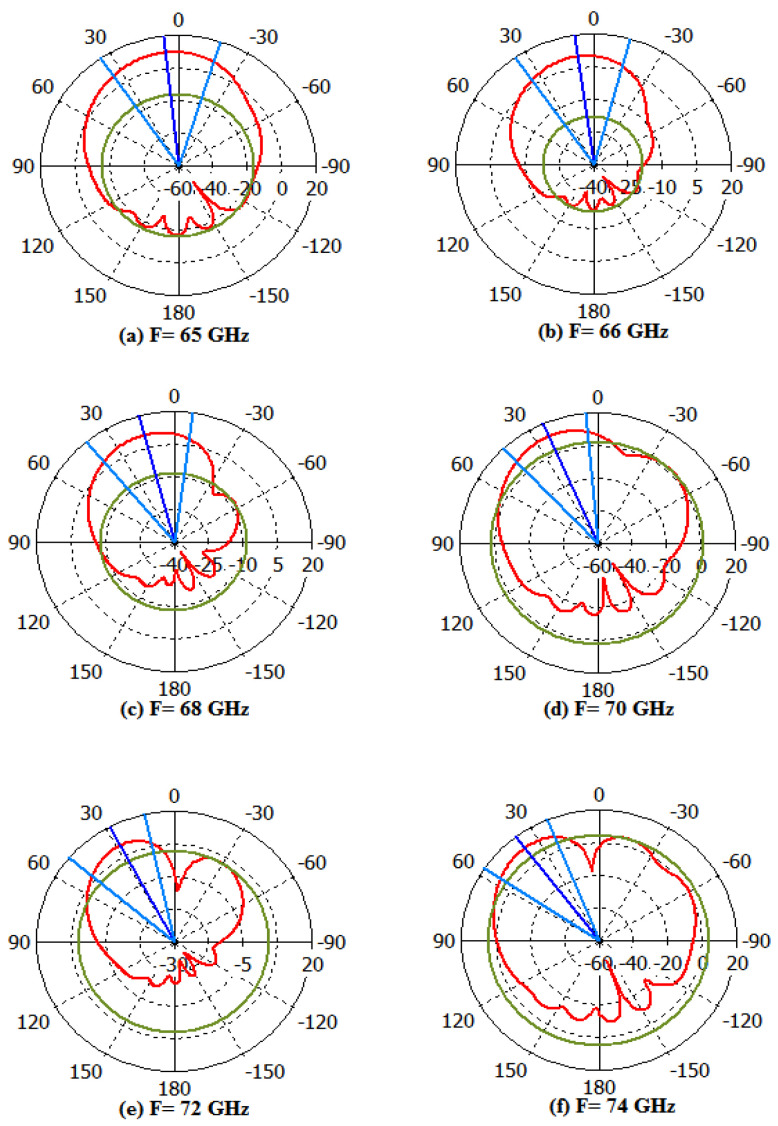
Far-field representation of Polar plot at (**a**) 65 GHz, (**b**) 66 GHz, (**c**) 68 GHz, (**d**) 70 GHz, (**e**) 72 GHz, (**f**) 74 GHz.

Figure 10 demonstrates the 2D far-field radiation behavior of gain at frequencies of 65, 66, 68, 70, 72, and 74 GHz, respectively. The angular width (3 dB) and main lobe magnitude and direction can be noticed in Figure 9. At 72 GHz, the angular width (3 dB) is 36.8°, and the main lobe direction is at 28°, whereas the maximum magnitude of the main lobe is 11.8 dB.

Table 2 compares the previously designed antenna with homogenous substrate and the antenna intended in this work with the heterogeneous substrate.

**Figure 11:**
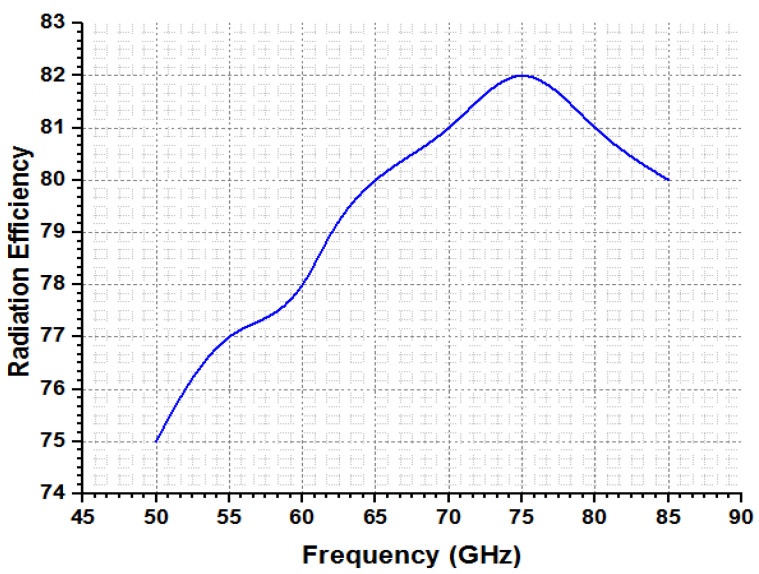
Radiation efficiency vs frequency estimations.

**Figure 12:**
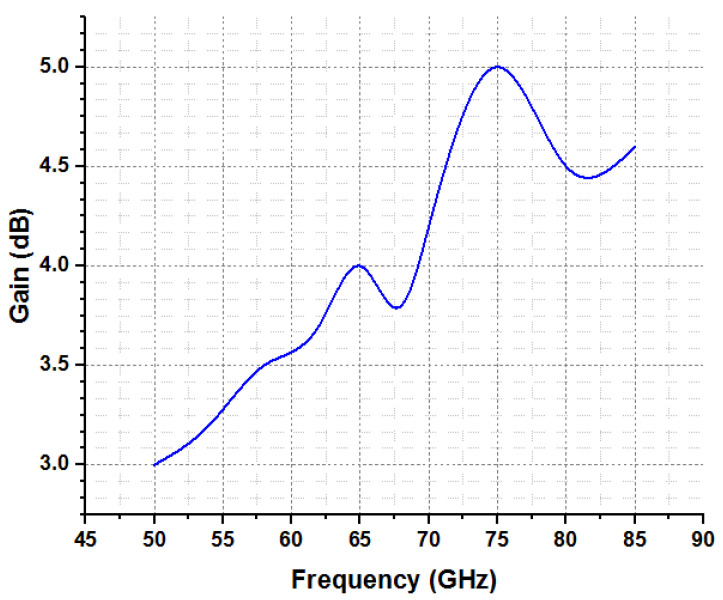
Investigation of gain as function of frequency.

The millimeter-wave spectrum, which is anticipated to be used in the next generation of 5G technology, is where the band of the intended antenna is located. With regard to applications such as device-to-device communication, WLAN, and WPAN, the V-Band (50–75 GHz) of millimeter waves should be used (D-2-D). The optimum range of the presented model is evaluated in Table 3, whereas Table 4 compares the proposed approach with state-of-the-art techniques, which explains that the outcomes of the presented model are more efficient than current models. The estimation among radiation efficiency and frequency is mentioned in Figure 11. This shows that the presented model efficiency is recorded at more than 85% at frequency ranges from 70 to 76 GHz. Additional investigations of the proposed model are performed in terms of gain and frequency, which are declared in Figure 12. It can be seen that the presented model exhibits fruitful gain at a frequency of 75 GHz.

## 5. Conclusions

For millimetre waves, a printed high-gain Yagi antenna was developed. The antenna’s straightforward design makes it easy to fabricate. The antenna is lightweight, low-cost, and low-profile. The planned antenna measures 10.8 × 10.01 mm^2^ in total. The antenna functions according to the conventional Yagi radiation theory. The developed antenna has a maximum return loss of −30 dB at 70.6 GHz and excellent impedance matching. It was found that as the electrical width of the effective aperture rises, so does the gain. How several of the parameters changed was examined, which had an impact on how gain improved. The heterogeneous substrate is another method that is used and is in charge of further improving gain, directivity, bandwidth, and efficiency. The developed antenna has an impedance bandwidth of 15.17 percent and 10.72 GHz. The antenna has a high gain of 11.8 dB and a high directivity of 12.1 dBi, respectively. The V-Band of mmW frequency spectrum, i.e., 50–75 GHz, is concentrated for the design’s band of operation. The 50–75 GHz frequency spectrum of mmW is undoubtedly planned in 5G intended for Device-to-Device (D-2-D) transmission. This is expected to be utilized in ad hoc communication around short distances. Furthermore, this spectrum could be used for high-frequency short-range WLAN, WHDMI, and WPAN.

## Figures and Tables

**Table 1:** Description of elements for designing antenna in mm.

SL	10	Lr	0.6
SW	10.7	Wr	2.2
Ld	1.6	S1	0.2
Wd	1.6	S2	1.3
Ld1 = Ld2	1.53	G	0.08
Wd1 = Wd2	1.1	h	0.11

**Table 2:** Results comparison of current and previous design.

Results	Gain (dBi)	Directivity (dB)	Bandwidth (GHz)	%Bandwidth	Efficiency (dB)
[20]	10.3	10.8	4.4	7.6	−0.88
Proposed model	12	12	11	15.2	−0.034

**Table 3:** Description of the designed model and achieved outcomes by presented model.

Parameter	Minimum Gain	Maximum Gain	Reflection Coefficient
Description	≥1.2 dB	≤3 dB	≤−10 dB
Optimum range achieved by proposed method	1.19 dB	2.89 dB	−10.14 dB

**Table 4:** Methodologies used by current models and proposed model.

Mechansims Used in Previous Models and Proposed Model	Machine-Learning-Based Models	Prescreening	Supervision	Number of Simulations
**GP-LCB**	GP	LCB	Nil	1990
**FBN-LCB**	BNN	LCB	Yes	1325
**BN-ALCB**	BNN	AdapLCB	Nil	1100
**GP-ALCB**	GP	AdapLCB	Nil	1260
**Presented model**	BNN	AdapLCB	Yes	930

## Data Availability

The data are available as per request.
